# Knowledge level regarding mpox among nurses in China: a cross-sectional study

**DOI:** 10.3389/fpubh.2026.1833332

**Published:** 2026-06-24

**Authors:** Yiting Xie, Wenqian Zhu, Zewei Chen, Yuelin Wu, Shiqing Liang, Minxia Hua, Xiaoli Yue, Jing Li, Jiahui Zhang, Xiangdong Gong

**Affiliations:** 1 Hospital for Skin Diseases, Institute of Dermatology, Chinese Academy of Medical Sciences & Peking Union Medical College, Nanjing, China; 2 Department of STD Epidemiology, National Center for STD Control, Nanjing, China; 3 Shanghai Pudong New Area Center for Disease Control and Prevention (Shanghai Pudong New Area Health Supervision Institute), Shanghai, China; 4 Wenzhou Lucheng Center for Disease Control and Prevention (Wenzhou Lucheng Health Inspection Institution), Zhejiang, China; 5 National Center for Cardiovascular Diseases, Beijing, China; 6 National Clinical Center of Cardiovascular Diseases, Fuwai Hospital, Chinese Academy of Medical Sciences, Beijing, China; 7 Beijing Haidian District Center for Disease Control and Prevention, Beijing, China

**Keywords:** China, cross-sectional study, knowledge level, mpox, nurses

## Abstract

**Background:**

Mpox poses a public health threat in China. Nurses are crucial in patient management, yet their mpox knowledge is poorly understood. This study aimed to describe mpox awareness among Chinese nurses, assess their level of mpox-related knowledge, and identify factors associated with good knowledge.

**Methods:**

An online cross-sectional survey was conducted with a self-designed questionnaire, collecting data on respondents’ demographics, clinical specialties, and institutional characteristics. Mpox knowledge was evaluated across etiology, clinical manifestations, transmission routes, and prevention and control measures, with a score of ≥30 out of 37 defined as good knowledge. Univariable and multivariable logistic regression analyses were performed to identify the influencing factors of good mpox knowledge.

**Results:**

Among 2,086 online questionnaires distributed to nurses across 19 Chinese provinces, 2,023 were valid (96.98% response rate). Among the 1,766 respondents who had heard of mpox and completed the knowledge assessment, 381 (21.57%) demonstrated good mpox knowledge. Domain-specific correct response rates were 52.71% for etiology, 68.47% for clinical characteristics, and 66.38% for transmission/prevention/control. Multivariable logistic regression showed that good mpox knowledge was positively associated with age >35 years, college degree or higher, employment in district/county-level hospitals, traditional media as knowledge source, and participation in mpox training.

**Conclusion:**

Mpox knowledge among Chinese nurses is inadequate. These findings highlight the urgent need for targeted, standardized, and multi-channel educational interventions, particularly for younger nurses, those in higher-level hospitals, and frontline clinical personnel. Strengthening professional training and improving access to reliable information sources are essential to enhance nurses’ preparedness and response capacity for emerging infectious diseases such as mpox.

## 1 Introduction

Mpox is a zoonotic disease caused by the mpox virus, which belongs to the genus Orthopoxvirus in the family Poxviridae (1). It is primarily found in the tropical rainforests of central and western Africa (2). Starting from early May 2022, multiple countries that are not endemic for mpox (mainly in Europe and Americas) reported mpox cases with no clear link to travel history in West Africa or Central Africa (3). Moreover, within a short period, the number of cases increased rapidly, affecting dozens of countries and regions. On July 23, 2022, following mpox outbreaks in various countries, the World Health Organization declared mpox a “public health emergency of international concern” (4, 5). On September 6, 2022, Hong Kong reported its first imported case of mpox (6). Ten days later, the first confirmed case of mpox in China was diagnosed in Chongqing Municipality (7). By August 2023, more than 1,000 domestic cases had been diagnosed in China. Hence, it is essential to remain vigilant about the mpox outbreak situation and implement comprehensive prevention and control measures to prevent its spread in China. Given the interconnectedness of global travel and trade, mpox outbreaks in any region can quickly spread across borders, highlighting the need for coordinated international responses and knowledge sharing among healthcare systems worldwide.

Nurses are at the frontline of mpox patient care, playing multifaceted roles in epidemic management. Their responsibilities include early identification of suspected cases during triage, implementation of isolation precautions, provision of supportive care to reduce complications (e.g., skin and mucosal lesions), patient education on transmission prevention, and psychological support to alleviate anxiety. However, some studies have revealed that healthcare workers may face occupational exposure to the mpox virus (8, 9). Therefore, specialized knowledge of mpox among nurses is essential for clinical diagnosis, treatment, and prevention of healthcare-acquired infections. Such knowledge can help prevent and control potential new mpox cases and related complications. Enhancing nurses’ mpox knowledge not only strengthens local outbreak control but also contributes to global health security by reducing the risk of international transmission through informed travel advice and early detection in border crossings.

Although several studies have investigated mpox knowledge among healthcare workers, most have focused on clinicians (10) or general healthcare workers (11, 12) rather than nurses specifically. In China, existing research has primarily targeted clinicians (13–15) or specific populations such as men who have sex with men (16, 17), leaving limited evidence on nurses’ mpox knowledge in China. Moreover, no study to date has comprehensively examined factors associated with nurses’ knowledge levels across different regions and hospital settings in China. Understanding these factors is crucial not only for national preparedness but also for informing global strategies, as lessons learned from China’s nursing workforce can provide valuable insights for other countries facing similar challenges in mpox prevention and control. Therefore, this study aimed to: (1) assess mpox awareness and knowledge levels among nurses across China; and (2) identify sociodemographic and professional factors associated with good mpox knowledge.

## 2 Methods

### 2.1 Study design and setting

A convenience sampling approach was used to recruit nurses currently in service from various levels of medical institutions across China. Participants were recruited from 19 provinces, autonomous regions, and municipalities in China through an online recruitment campaign conducted via the STD Prevention and Control New Media Health Communication and Service Platform and Control New Media Health Communication and Service Platform (Hand in Hand Medical Visiting) between September 16 and 29, 2022. The research protocol obtained ethical approval from the Institutional Review Board of the Institute of Dermatology, Chinese Academy of Medical Sciences (Approval Number: 2022-KY-042).

Inclusion criteria were: (1) holding a valid Chinese nursing practice certificate; (2) currently employed as a clinical nurse in a medical institution in China; (3) voluntarily participated in the study and provided informed consent.

Exclusion criteria were: (1) nursing interns or nurses undergoing advanced or standardized training programs; (2) nursing staff engaged in administrative or logistics positions without clinical nursing work during the survey period; (3) respondents with obviously inconsistent or logically contradictory responses in the questionnaire.

### 2.2 Study instrument: mpox knowledge questionnaire

#### 2.2.1 Questionnaire design

The questionnaire was devised based on authoritative references, including the Interim Rapid Response Guidelines for Mpox (WHO) (18), Mpox Diagnostic and Treatment Guidelines (2022 Edition) (19), and Mpox Prevention and Control Technical Guidelines (2022 Edition) (19).

The structured questionnaire encompassed three key dimensions: the etiology of mpox, clinical characteristics, and transmission, prevention, and control. The knowledge assessment consisted of 37 items covering three domains: etiology, clinical characteristics, and transmission, prevention, and control. Additional sections collected demographic, professional, and institutional characteristics. Each question was assigned 1 point, yielding a total possible score of 37 points. The questions incorporated multiple-choice items for which respondents were required to select at least one correct option and refrain from choosing any incorrect options to receive a correct-answer score. The scoring scheme was as follows: 8 points were allocated for questions related to mpox etiology, 15 points for those on clinical characteristics, and 14 points for questions concerning transmission, prevention, and control. To define good knowledge, a modified Bloom’s cutoff point of 80% (≥30/37) was applied. This criterion is widely used in public health education research to indicate mastery of critical concepts when no gold standard exists. Several studies (15, 16, 20, 21) on healthcare workers’ knowledge of emerging infectious diseases, including mpox and COVID-19, have adopted similar thresholds to distinguish between good and inadequate knowledge. The 80% threshold ensures that participants demonstrate strong comprehension of essential information necessary for safe clinical practice and effective public health response. It also allows comparability across studies, supporting meta-analytic assessments of healthcare workforce preparedness. Participants who reported that they had never heard of mpox were excluded from the knowledge-score analysis because they did not complete the detailed mpox knowledge assessment. In this study, awareness referred only to whether participants had previously heard of mpox, whereas knowledge referred to the scored understanding of mpox assessed using the 37-item questionnaire. Among participants who had heard of mpox and completed the knowledge questionnaire, good knowledge was defined as a total score of ≥30 out of 37, whereas a score of <30 was classified as inadequate knowledge.

#### 2.2.2 Sample size estimation

The sample size was calculated using the standard sample size formula for cross-sectional studies: $n=\frac{z_{\alpha /2}^{2}p(1-p)}{\delta^{2}}$ . Based on previous similar studies (15, 22), the estimated proportion with good mpox knowledge (*p*) of nursing staff was set at 50.0% (to maximize the sample size), with an allowable error (*δ*) of 0.05 and a two-tailed test level (*α*) of 0.05 (*Z*_*α*/2​_ ≈ 1.96). The sample size was estimated as 402 cases based on the study parameters. Considering a potential invalid questionnaire rate of 10–20% for online surveys and the requirements of multivariable analysis, the target sample size was expanded to at least 500 cases. A total of 2023 valid samples were finally collected, which met the statistical analysis requirements.

#### 2.2.3 Survey regions

China was divided into six geographic regions for this study: North China: Beijing, Tianjin, Hebei Province, Shanxi Province, Inner Mongolia Autonomous Region. Northeast China: Liaoning Province, Jilin Province, Heilongjiang Province. East China: Shanghai, Jiangsu Province, Zhejiang Province, Anhui Province, Fujian Province, Jiangxi Province, Shandong Province. Central and South China: Henan Province, Hubei Province, Hunan Province, Guangdong Province, Guangxi Zhuang Autonomous Region, Hainan Province. Northwest China: Shaanxi Province, Gansu Province, Qinghai Province, Ningxia Hui Autonomous Region, Xinjiang Uygur Autonomous Region. Southwest China: Chongqing Municipality, Sichuan Province, Guizhou Province, Yunnan Province, Xizang Autonomous Region.

#### 2.2.4 Reliability and validity of the questionnaire

As a self-designed instrument, the questionnaire was developed without a previously validated translated version; instead, content validity was established through expert review by five specialists in infectious disease, public health, and nursing, and items were revised iteratively until consensus was reached. The internal consistency of the knowledge scale was assessed using Cronbach’s alpha coefficient, which yielded a value of 0.95 for the overall scale (etiology subscale: 0.80; clinical characteristics subscale: 0.93; transmission and control subscale: 0.89), indicating acceptable to good reliability. Construct validity was evaluated using the Kaiser–Meyer–Olkin (KMO) measure and Bartlett’s test of sphericity. The KMO value for the overall scale was 0.96 (etiology subscale: 0.86; clinical characteristics subscale: 0.96; transmission and control subscale: 0.90), and Bartlett’s test was significant for all subscales and the total scale (all *p* < 0.01), confirming the suitability of the data for factor analysis. All scoring procedures followed the item-level binary scoring rules described above, consistent with approaches used in prior mpox knowledge surveys among healthcare workers.

### 2.3 Data processing and analytic strategy

To ensure the quality and reliability of the questionnaire responses, several measures were implemented. First, mandatory fields and logical skip patterns were incorporated into the survey design to prevent incomplete or inconsistent answers. Additionally, technical restrictions were applied to limit each mobile phone number to a single submission, thereby eliminating the possibility of duplicate entries by the same individual. These controls were established to maintain data integrity and minimize potential biases in the study results.

Because participants who had never heard of mpox did not complete the detailed knowledge assessment, the knowledge-score analysis and multivariable logistic regression were restricted to participants who had heard of mpox and completed the 37-item knowledge questionnaire. Awareness was reported descriptively among all valid respondents, thereby separating prior awareness from the depth of mpox knowledge.

### 2.4 Statistical analysis

Descriptive statistics were used to characterize the demographic and professional characteristics of the participants. Normally distributed quantitative data were expressed as mean ± standard deviation (X ± S), and non-normally distributed quantitative data were presented as median with interquartile range (M [Q1, Q3]). Categorical data were summarized as rates or constituent ratios (%).

Multivariable logistic regression analysis was performed with mpox knowledge level as the dependent variable, dichotomized as good knowledge versus inadequate knowledge according to the predefined cutoff score of ≥30 out of 37. Independent variables with a *p* value ≤ 0.25 (23) in the Univariable analysis were included in the multivariable regression model. Multicollinearity was assessed using variance inflation factor (VIF), with VIF values <5 considered to indicate no significant collinearity. A two-tailed test with a significance level (*α*) of 0.05 was used for all hypothesis tests. Data were collated and cleaned using Microsoft Excel 2016, and all statistical analyses were conducted with SPSS 18.0 software. Responses with missing data in the knowledge assessment section were excluded from the analysis. The number of such exclusions was minimal and is reported in the results. Model goodness-of-fit was assessed using the Hosmer–Lemeshow test.

## 3 Results

### 3.1 Sociodemographics and professional characteristics of the participants

**Figure 1:**
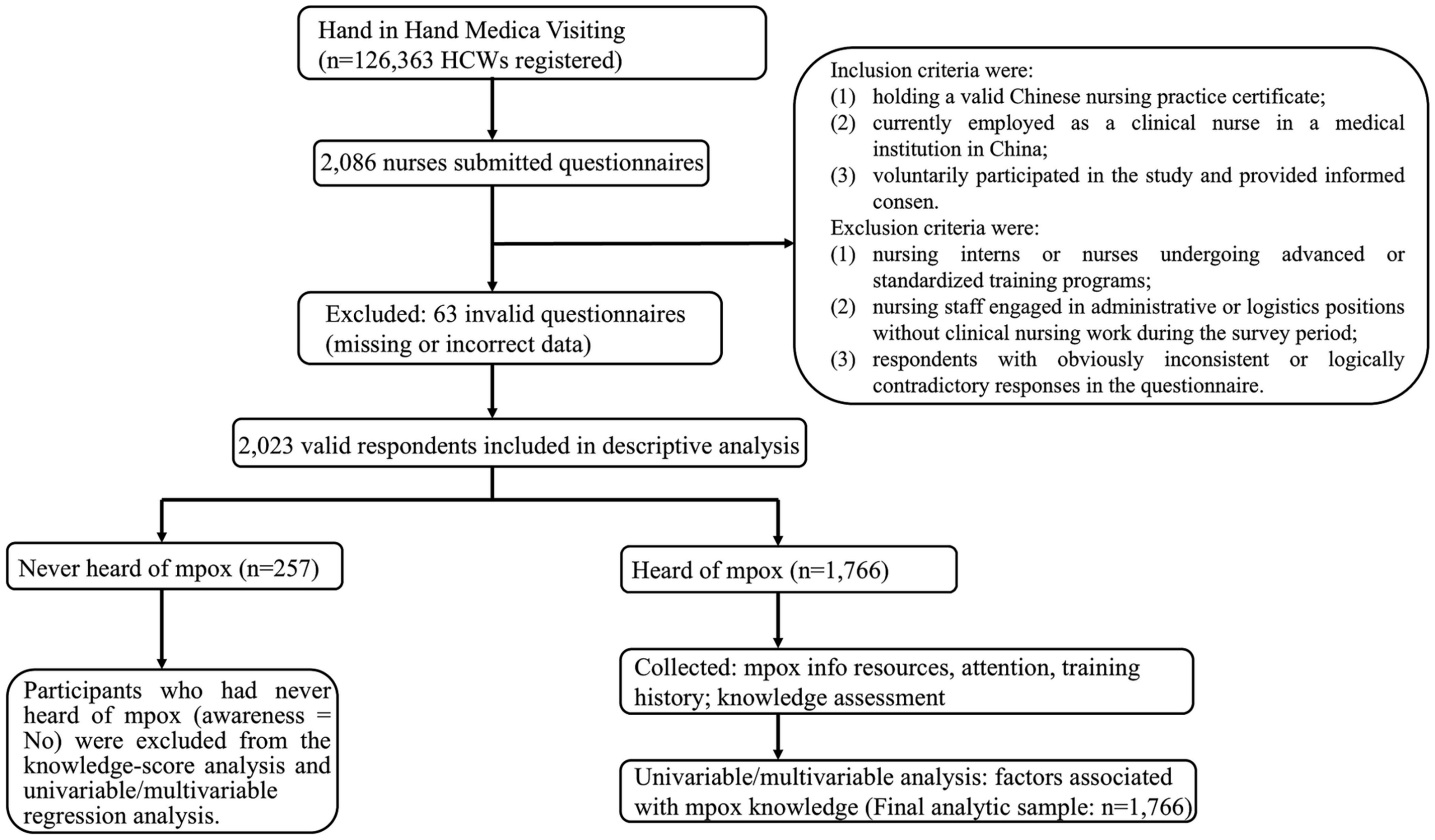
Flowchart of participant selection and analytic sample construction. A total of 2,086 nurses submitted questionnaires. After excluding 63 invalid questionnaires, 2,023 valid respondents were included in the descriptive analysis. Among them, 1,766 participants had heard of mpox and completed the detailed knowledge assessment, whereas 257 participants had never heard of mpox and were excluded from the knowledge-score analysis and multivariable logistic regression. The final analytic sample for multivariable logistic regression was 1,766.

A total of 2,086 online questionnaires were collected from nursing staff across 19 provinces (autonomous regions and municipalities) in China, with 2,023 valid responses, yielding a validity rate of 96.98%. The 63 excluded questionnaires comprised 40 with logically inconsistent answers (e.g., conflicting responses to similar items) and 23 with incomplete responses in the mpox knowledge assessment section (Figure 1).

The participants had a mean age of (32.62 ± 8.32) years, ranging from 19 to 60 years. Married participants accounted for 68.76% (1,391/2,023) of the total sample. In terms of educational background, the majority held a college degree or above, representing 93.67% (1,895/2,023). Regarding professional titles, 62.97% (1,274/2,023) held junior titles, while 37.03% (749/2,023) possessed intermediate or higher titles.

### 3.2 Department and medical institution characteristics

In terms of clinical departments, 9.34% (189/2,023) of participants worked in dermatology/STD or infectious diseases departments, 16.96% (343/2,023) in gynecology/pediatrics departments, and the remaining 73.70% (1,491/2,023) in other clinical departments. For medical institution levels, 68.61% (1,388/2,023) were affiliated with district/county-level hospitals, and 31.39% (635/2,023) with municipal-level or higher hospitals. Based on hospital grades, 69.15% (1,399/2,023) worked in Grade II or below hospitals, and 30.85% (624/2,023) in Grade III hospitals. Data completeness was high for all variables included in the analysis, with no missing values observed in the final dataset of 2,023 participants.

### 3.3 Attention to mpox-related information in the past 6 months

Regarding the level of attention to mpox-related information over the past 6 months, 27.68% (560/2,023) of participants reported frequent attention, 48.84% (988/2,023) paid occasional attention, and 23.48% (475/2,023) showed rare or no attention to such information.

### 3.4 Sources of mpox knowledge acquisition

The main sources of mpox knowledge acquisition among participants were summarized as follows: 50.82% (1,028/2,023) obtained relevant knowledge through traditional media channels (television, newspapers, radio), and 81.41% (1,647/2,023) via new media platforms (WeChat, Weibo, Douyin). Additionally, 57.54% (1,164/2,023) of the nursing staff reported having participated in specialized mpox knowledge training.

### 3.5 Knowledge level of mpox

Of the 2,023 valid respondents, 1,766 had heard of mpox and completed the knowledge assessment. 381 (21.57%) were classified as having good mpox knowledge, whereas 1,385 (78.43%) had inadequate knowledge. Nurses demonstrated better knowledge levels regarding the clinical characteristics, transmission and control of mpox than in the area of its etiology.

(1) Knowledge level of mpox etiology

The correct response rate for the etiology domain was 52.71%. Most participants correctly identified effective methods for inactivating the mpox virus (82.96%), whereas only 42.92% correctly knew that mpox virus has two branches: West Africa and Congo Basin (Table 1).

(2) Knowledge level of the clinical characteristics of mpox

**Table 1:** Description of the responses to the items regarding the etiological knowledge of mpox.

Knowledge questions	Correct (%)
1. Mpox virus is a double-stranded DNA virus.	48.02%
2. Mpox virus can be cultured *in vitro*.	54.53%
3. The mpox virus belongs to the genus Orthopoxvirus in the family Poxviridae together with the smallpox virus, cowpox virus, and poxvirus, and its infectivity and pathogenicity are weaker than that of smallpox.	52.38%
4. There is cross-immunity between mpox virus and smallpox virus.	56.63%
5. Mpox virus and chickenpox virus are not in the same family.	45.53%
6. Two clades: the West African clade and the Congo Basin clade.	42.92%
7. Suitable conditions for the survival of mpox virus.	38.73%
8. Effective methods of inactivation of mpox virus.	82.96%

The correct response rate for the clinical characteristics domain was 68.47%. The vast majority (89.01%) correctly responded to the clinical symptoms of mpox infection in the prodromal stage, but the minority (22.25%) accurately knew the criteria for determining confirmed cases of mpox (Table 2).

(3) Knowledge level of the transmission, prevention, and control of mpox.

**Table 2:** Description of the responses to the items regarding the clinical characteristics of mpox.

Knowledge questions	Correct (%)
1. The usual incubation period of mpox virus.	40.60%
2. The shortest and longest incubation periods of mpox virus.	41.90%
3. The clinical signs of the prodromal phase of mpox infection.	89.01%
4. The mpox rash undergoes several stages of change from macules, papules, vesicle, pustules and crusts, with crusts appearing in about 10 days.	71.57%
5. Characteristics of mpox rash.	34.31%
6. The main sites of mpox rash.	84.48%
7. Differential diagnosis of mpox.	83.47%
8. Laboratory methods of mpox.	84.37%
9. Criteria of mpox suspected cases.	75.99%
10. Criteria for determining confirmed cases of mpox.	22.25%
11. Before treating a patient with mpox, the patient should be asked if he/she is infected with HIV.	78.03%
12. At present, there is no specific anti-mpox virus drug in China, mainly for symptomatic support and prevention of complications.	79.05%
13. Complications that may occur in patients with severe cases of mpox.	83.75%
14. High-risk groups for mpox complications.	87.54%
15. Mpox can cause death in patients.	82.05%

The correct response rate for the transmission, prevention, and control domain was 66.38%. Although most participants correctly identified the susceptible groups of mpox, only a minority correctly identified the transmission route of mpox (Table 3). Notably, the survey was conducted shortly after China’s first mpox case was reported, which may have underestimated nurses’ knowledge levels as relevant training had not yet been widely implemented. This timing factor should be considered when interpreting the overall knowledge status observed in this study.

**Table 3:** Description of the responses to the items regarding the transmission and control of mpox.

Knowledge questions	Correct (%)
1. Mpox is a zoonosis.	87.66%
2. The main host of mpox virus.	49.21%
3. The source of mpox virus infection.	67.84%
4. Mpox infection can be divided into two phases: the prodromal phase and the rash phase, and the rash phase is more contagious.	54.30%
5. The transmission route of mpox.	19.20%
6. The contact mode of spreading mpox.	89.13%
7. The susceptible group of mpox.	92.19%
8. The preventive measures of mpox.	92.13%
9. Smallpox vaccination is also needed for mpox vaccination.	70.22%
10. Mpox vaccine is now available in the United States.	43.77%
11. People who can be vaccinated against mpox in the United States.	41.22%
12. Your advice for preventing and treating mpox if a close friend or a person with mpox seeks your help.	91.85%
13. What you would do if you came into contact with a mpox carrier or a person with mpox.	91.96%
14. If you find a suspected or confirmed case of mpox, you should report it directly through the surveillance and report management module of the China Information System for Disease Control and Prevention (CISDPC) within 24 h.	39.58%

### 3.6 Results of statistical analysis

Based on the previously described scoring approach, participants with a score ≥30 out of 37 were considered to have a good level of mpox knowledge, and participants with a score <30 had inadequate mpox knowledge. The results of the univariable analysis identified several variables with a *p* value < 0.25, which were considered for inclusion in the multivariable logistic regression analysis, including age group, professional title, hospital level, hospital grade, attention to mpox-related information, mpox knowledge acquisition via traditional media, and participation in mpox training. However, region and department were not entered into the final model because they were multi-category variables with uneven subgroup distributions and relatively small numbers of outcome events in some categories, which could have resulted in unstable estimates.

Multicollinearity diagnosis indicated that all variance inflation factors (VIF) were less than 5 (range: 1.02–1.76), suggesting no significant multicollinearity among the independent variables. The final model showed a good fit, as indicated by the Hosmer–Lemeshow *χ*^2^ = 4.30 (*p* = 0.74 > 0.05) and a Nagelkerke *R*^2^ of 0.15. Forward conditional multivariable analysis was performed, and the results indicated that being older than 35 years (OR = 1.58, 95% CI: 1.22–2.04, *p* < 0.001), having a college degree or higher (OR = 1.90, 95% CI: 1.10–3.29, *p* = 0.022), employment in district/county-level medical institutions (OR = 2.11, 95% CI: 1.61–2.77, *p* < 0.001), knowledge acquisition via traditional media (OR = 1.44, 95% CI: 1.13–1.86, *p* < 0.001), and participation in mpox training (OR = 5.12, 95% CI: 3.65–7.17, *p* < 0.001) were positively associated with a good level of mpox knowledge among nurses (Table 4).

**Table 4:** Factors associated with good mpox knowledge among nurses (*n* = 1,766).

Variables	Respondents		Good know-ledge	Good know-ledge (%)	Univariable analysis		Multivariable analysis	
					OR (95% CI)	*p*	*OR*(95%*CI*)	*p*
Age group	≤35 years	1,260	248	19.68	1	–	–	–
	>35 years	506	133	26.28	1.46 (1.14–1.85)	0.002	1.58 (1.22–2.04)	<0.001
Marital status	Divorced/widowed	58	10	17.24	1		–	–
	Married	1,205	269	22.32	1.38 (0.69–2.76)	0.36	–	–
	Unmarried	503	102	20.28	0.94 (0.60–2.50)	0.58	–	–
Region	North China	73	15	20.55	1		–	–
	Northeast China	743	138	18.57	0.88 (0.49–1.60)	0.680	–	–
	East China	226	84	37.17	2.29 (1.22–4.29)	0.010	–	–
	South China	701	139	19.83	0.96 (0.53–1.74)	0.884		
	Northwest China	12	4	33.33	1.93 (0.51–7.29)	0.330		
	Southwest China	11	1	9.09	0.39 (0.05–3.26)	0.383		
Education level	High school or below	115	17	14.78	1		–	–
	College or above	1,651	364	22.05	1.63 (0.96–2.74)	0.070	1.90 (1.10–3.29)	0.022
Professional title	Junior	1,107	212	19.15	1		–	–
	Intermediate or above	659	169	25.64	1.46 (1.16–1.83)	<0.001	–	–
Department	Other departments^a^	1,289	286	22.19	1		–	–
	Gynecology/pediatrics	310	56	18.06	0.77 (0.56–1.06)	0.113	–	–
	Dermatology/infectious Diseases	167	39	23.35	1.07(0.73–1.57)	0.734	–	–
Hospital level	Municipal or above	572	88	15.38	1		–	–
	District/county	1,194	293	24.54	1.79 (1.38–2.33)	<0.001	2.11 (1.61–2.77)	<0.001
Hospital Grade	Tertiary	559	109	18.20	1		–	–
	Secondary or below	1,207	272	22.54	1.20 (0.95–1.54)	0.149	–	–
Attention to mpox-related information in the past 6 months								
Rarely		218	21	9.63	1		–	–
Occasionally		988	198	20.04	2.25 (1.46–3.79)	<0.001	–	–
Frequently		560	162	28.93	3.82 (2.35–6.21)	<0.001	–	–
Acquisition of mpox knowledge via new media^b^								
No		119	21	17.65	1		–	–
Yes		1,647	360	21.86	1.31 (0.80–2.12)	0.282	–	–
Acquisition of mpox knowledge via traditional media^c^								
No		738	121	16.40	1		–	–
Yes		1,028	260	25.29	1.73 (1.36–2.20)	<0.001	1.44 (1.13–1.86)	<0.001
Participated in training								
No		602	44	7.31	1		–	–
Yes		1,164	337	28.95	5.17 (3.71–7.02)	<0.001	5.12 (3.65–7.17)	<0.001

This independent variable was not included in the multivariable analysis or has been excluded by the forward method.

a Other departments included gastroenterology, neurology, endocrinology, orthopedics, and emergency medicine.

b New media (WeChat, Weibo, Douyin).

c Traditional media (television, newspapers, radio).

## 4 Discussion

Among respondents who had heard of mpox and completed the knowledge assessment, only 21.57% achieved a good knowledge level, indicating inadequate mpox knowledge. This finding differs from that reported by Huang et al. (22), which may be partly explained by differences in study populations, questionnaire design, scoring criteria, and survey timing. In comparison, 16.9% of nurses in Saudi Arabia demonstrated adequate knowledge regarding mpox (24), a proportion slightly lower than that observed in the present study. Differences across studies may be attributable to variations in assessment tools, cutoff values, sample size, professional background, and the stage of the mpox outbreak at the time of data collection. Furthermore, the level of mpox knowledge among nurses in this study was comparatively lower than that reported in studies focusing on clinicians. Findings from research conducted in China (15), Lebanon (12), Egypt (25), Turkey (10), and Kuwait (26) consistently showed that clinicians were more likely to have a high level of knowledge about mpox compared to nurses. This disparity may be attributed to distinct clinical roles and responsibilities within healthcare teams: clinicians primarily concentrate on the diagnosis and treatment of mpox (27), while nurses are more focused on patient care and the prevention of complications associated with the disease.

Age was significantly associated with knowledge level, with nurses age >35 years demonstrating higher knowledge scores. This finding is consistent with previous studies (26) showing that older healthcare workers tend to have better mpox-related knowledge than younger healthcare workers. This may reflect the cumulative effect of clinical experience and prolonged exposure to infectious disease training. More experienced nurses may have greater familiarity with emerging infectious diseases and stronger professional knowledge, which facilitates knowledge acquisition. However, given the cross-sectional design, causality cannot be established.

Educational level was another important determinant, with nurses holding a college degree or higher showing better knowledge. This finding is consistent with previous studies (28, 29) indicating that higher education enhances individuals’ ability to understand, process, and apply medical information. It suggests that differences in baseline educational background may contribute to disparities in knowledge acquisition among healthcare workers.

In the univariable analysis, nurses working in secondary or below hospitals had a higher proportion of good mpox knowledge; however, hospital grade was not independently associated with knowledge level after adjustment. By contrast, employment in district/county-level medical institutions remained positively associated with good knowledge in the multivariable model.

The study results showed that nurses working in district/county-level medical institutions had a higher likelihood of good mpox knowledge, which was inconsistent with previous domestic research findings (13). This may be related to China’s early mpox response strategy, in which public health training and risk communication were rapidly disseminated through the Centers for Disease Control and Prevention (CDC) networks and primary healthcare systems, often reaching district- and county-level institutions earlier. In contrast, nurses in municipal-level or higher hospitals may have faced heavier clinical workloads, limiting their participation in early training.

In addition, knowledge acquisition via traditional media was positively associated with better knowledge levels (28). This may reflect the broad accessibility and credibility of traditional media channels such as television and official news outlets, particularly in the early stages of emerging infectious disease outbreaks. Compared with new media, traditional media may provide more standardized and authoritative information, which is crucial for healthcare workers. This finding suggests that conventional information dissemination channels remain important in public health education. Participation in mpox-related training showed the strongest association with knowledge level, indicating that structured training programs play a critical role in improving mpox knowledge (28). This finding underscores the importance of institutionalized and regular training mechanisms for emerging infectious diseases. Although causal inference cannot be drawn, the strength of the association suggests that training is a key modifiable factor that can be targeted in intervention strategies. Recent evidence (30) has further emphasized the need for integrated educational and behavioral interventions to improve healthcare workers’ preparedness for mpox.

In the analysis of knowledge related to the clinical presentation of mpox, the knowledge scores of nurses regarding the diagnostic criteria for confirmed cases of mpox were relatively low, only 22.25% correctly identified the criteria for confirmed mpox cases. This knowledge gap is clinically important because uncertainty about confirmation criteria may delay triage, laboratory testing, isolation, referral, and statutory case reporting, thereby increasing the risk of missed or delayed diagnosis and potential nosocomial transmission. Several previous small-scale studies have also found insufficient understanding among healthcare workers regarding diagnosis and treatment, while the efficiency of medical staff in screening and diagnosing cases of mpox has an impact on the economic burdens borne by patients, their families, and society (31, 32). Furthermore, the accuracy rate of knowledge questions regarding mpox transmission routes among Chinese nurses remained sub-optimal. Previous studies have indicated that healthcare workers are at risk of occupational exposure to the mpox virus (8, 9), with less experienced nurses facing a particularly heightened risk (33). Consequently, the level of knowledge among nurses regarding mpox transmission routes may significantly impact both the clinical management of mpox patients and the prevention of healthcare-associated infections. These findings underscore the need to prioritize targeted educational interventions to enhance knowledge of mpox transmission mechanisms and infection control measures.

Recent studies have confirmed that the current mpox outbreaks are primarily transmitted through sexual contact among men who have sex with men (MSM) (1, 17). The early stage of the disease typically manifests with symptoms such as fever and headache (2). Following the febrile phase, patients may develop complications including characteristic skin and mucous lesions (2, 3). Consequently, the primary departments involved in diagnosis and management are the Department of Infectious Diseases and the Department of Dermatology (34, 35). However, our study detected no significant difference in the mpox-specialized knowledge between the nurses in the aforementioned departments and those in other departments, which is contrary to the results of national studies (13, 36). This finding may be partly explained by the relatively small proportion of nurses from specialized departments in our sample (9.34%), which may have limited the statistical power to detect between-department differences. In addition, the survey was conducted during the early stage of the mpox outbreak in China, when department-specific training had not yet been fully implemented. As a result, knowledge levels may not yet have diverged across departments. Since supportive care of mpox patients helps to reduce skin and mucosal complications (37), healthcare organizations need to strengthen the training of mpox expertise in departments such as infectious diseases, dermatology, and other relevant departments. Future studies with larger samples from specialized departments are warranted.

This study has several limitations. First, the cross-sectional design precludes causal inference; therefore, the observed associations should be interpreted as correlations and may be influenced by unmeasured confounders, such as institutional training policies and individual learning motivation. Second, the use of convenience sampling through an online platform may have introduced selection bias. The survey was conducted via the “Hand in Hand Medical Visiting” platform, which primarily targets healthcare professionals. Users of this platform are more likely to have access to digital health information and professional training resources, and may therefore have higher baseline knowledge or interest in infectious diseases. As a result, nurses with greater engagement in health information or digital tools may have been overrepresented, potentially leading to an overestimation of knowledge levels and limiting the generalizability of the findings. Although participants were recruited from 19 provinces, caution is still needed when extrapolating the results to the broader nursing population in China. Third, mpox knowledge was assessed using a self-designed questionnaire. Although reliability and validity were acceptable, self-reported data remain subject to recall and social desirability bias. Additionally, the 80% cutoff used to define good knowledge (≥30/37), while widely applied, is somewhat arbitrary and may lead to loss of information. Finally, the survey was conducted at the early stage of the mpox outbreak in China, prior to widespread training; therefore, the findings likely reflect baseline knowledge rather than current preparedness following subsequent educational interventions.

## 5 Conclusion

This study demonstrates that mpox-related knowledge among nurses in China remains inadequate, with notable gaps in etiology and transmission routes. Knowledge levels were significantly associated with age, educational level, hospital level, information sources, and participation in training. Among these, structured training and access to reliable information sources emerged as key modifiable factors. These findings highlight the need for targeted and standardized educational interventions, particularly for younger nurses, those in higher-level hospitals and frontline clinical personnel. Training programs should prioritize critical knowledge areas, including diagnostic criteria and transmission mechanisms. In addition, a multi-channel dissemination strategy integrating traditional media, new media, and institutional training should be adopted to improve both accessibility and effectiveness of knowledge delivery. Strengthening continuous education for nurses will enhance clinical preparedness and infection control capacity, supporting effective responses to emerging infectious diseases such as mpox.

## Data Availability

The original contributions presented in the study are included in the article/Supplementary material, further inquiries can be directed to the corresponding author.
